# Reproductive and morphological traits distinguish early- and late-maturing Pacific lamprey (*Entosphenus tridentatus*)

**DOI:** 10.1007/s00360-026-01692-x

**Published:** 2026-07-20

**Authors:** James J. Nagler, Lea R. Medeiros, Jon E. Hess, Tod M. Sween, Laurie L. Porter

**Affiliations:** 1 Department of Biological Sciences, University of Idaho, 875 Perimeter Dr MS 3051, Moscow, ID 83844 USA; 2 Columbia River Inter-Tribal Fish Commission, 700 NE Multnomah Street, Suite 1200, Portland, OR 97232 USA; 3 Nez Perce Tribe Department of Fisheries Resources Management, P.O. Box 365, Lapwai, ID 83540 USA

**Keywords:** Fish, Life history, Phenotype, Sex, Estradiol

## Abstract

Pacific lamprey, an anadromous fish native to the North Pacific Ocean, are imperiled in parts of their range. Many aspects of their biology are not fully understood, including the physiology of their spawning migration. Pacific lamprey exhibit two divergent reproductive life histories: early-maturing and late-maturing. Interior Columbia River early-maturing lamprey complete the final stages of gonadal maturation ~ 10 months after entering freshwater. From the same migrating cohort, the late-maturing lamprey wait ~ 22 months to complete maturation. This study sought to characterize the reproductive phenotypes of these two Pacific lamprey life histories. Migrating adult Pacific lamprey that had entered fresh water were collected and held for close to two years to monitor changes in morphology and physiology. The lamprey in this study, of approximately equal sex ratio, clearly separated into early- or late-maturing life histories based on differences in timing of reproductive development, morphology of length, weight, interdorsal fin gap, and plasma concentrations of estradiol-17β (E2), but not plasma androstenedione. Average plasma E2 levels in females and males were significantly higher in the early-maturing fish compared to the late-maturing fish by November, nearly half a year before any morphological divergence. This novel physiological indicator could benefit restoration activities (translocation and artificial propagation) by providing advanced identification of the two maturation life histories present in the same migration cohort. In conclusion, this study provides several morphological and physiological features that can be used to identify early- and late-maturing Pacific lamprey within the 10 month period prior to when the early-maturing fish mature.

**Supplementary Information:**

The online version contains supplementary material available at 10.1007/s00360-026-01692-x.

## Introduction

The Pacific lamprey (*Entosphenus tridentatus*) is an anadromous fish whose native range extends from Japan to California in the northern Pacific Ocean. The lifecycle of this fish begins in freshwater streams and rivers for several years, followed by metamorphosis and migration to the ocean for a marine residence period of 4–7 years where they predate other fishes and marine mammals, before they return to freshwater to spawn and die (Beamish 1980; Hess et al. 2022; Potter et al. 2015). Pacific lamprey are significant ecosystem contributors in both freshwater and marine environments (Beamish 1980; Close et al. 2002) and are culturally important to Indigenous people living in the North American Pacific region (Close et al. 2004). Pacific lamprey are imperiled in many parts of their range (CRITFC 2025) and management activities such as hatchery propagation (Lampman et al. 2021) and translocation (Hess et al. 2022; Ward et al. 2012) are being applied to restore the population to historical levels of abundance within the Columbia River Basin.

The reproductive biology of lampreys is, in general, understood (Docker et al. 2019). They are semelparous egg-layers (i.e., oviparous) with external fertilization. Anadromous lamprey do not feed upon entering fresh water to spawn; all energy for locomotion, building gonads, and spawning activities is derived from stored lipid, protein and carbohydrate (Beamish et al. 1979). The reproductive endocrinology of lampreys is similar in most respects to bony fishes (Sower 2003; Sower and Kawauchi 2001), except for the sex steroids. In contrast to bony fishes, which exhibit high levels of the principal circulating estrogen (estradiol-17β; E2) in females, E2 in lampreys is found at similar levels in maturing individuals of both sexes (Docker et al. 2019). In fact, male Pacific lamprey can have higher levels of plasma E2 than females during the 9 months leading up to final maturation (Mesa et al. 2010), similar to sea lamprey (Linville et al. 1987). This is distinctly different from the lifecycle of male bony fishes studied to date, whose plasma estrogens levels are always much lower than in females – often near the limit of detection (Fostier et al. 1983). Instead, male bony fishes utilize androgens to facilitate reproductive development (Fostier et al. 1983). The importance of androgens in reproductively active lampreys has been examined, and some 15-hydroxylated androgens have been implicated in male lampreys of several species (Bryan et al. 2006), with a receptor specific for androstenedione in the sea lamprey (*Petromyzon marinus*) testis having been reported (Bryan et al. 2007). However, a defined role for androstenedione in the male or seasonal plasma concentrations of androstenedione in maturing individuals of either sex have not been reported. The lack of clearly defined, sexually dimorphic roles for circulating sex steroids could explain why there is no external sexual dimorphism in Pacific lamprey until a month or two before spawning.

Life history diversity is a hallmark of lampreys and is thought to be an important factor contributing to their persistence and survival (Clemens et al. 2013, 2026; Hess et al. 2013). For Pacific lamprey entering fresh water to spawn, intraspecific diversity has been reported for phenotypes differing in appearance, migration timing, and the state of gonad development. There are historical reports from the Columbia River, WA that upriver migrating Pacific lamprey vary in size and coloration and could be roughly separated into two groups: larger grey/blue individuals and smaller, brown-colored individuals (Close et al. 2004). The smaller, brown Pacific lamprey were thought to have resided in the river longer than the larger grey/blue individuals, potentially for a year or more. More recent work has determined a genetic basis for these differences, indicating a correlation between large body size and migration distance (Hess et al. 2014). Run timing can also vary within the same watershed. Pacific lamprey in the Russian River, CA have two different periods of spawning migration each year – a large spring migration and a smaller fall migration (Moyle et al. 2009). Pacific lamprey returning to spawn in the Klamath River, CA and Willamette River, OR have been characterized by two reproductive life histories, “ocean-maturing” and “stream-maturing" (Clemens et al. 2013). The ocean-maturing life history variant is represented by fish entering fresh water with gonads significantly larger than the stream-maturing counterpart. Follow-up work demonstrated a genetic basis for ocean- and stream-maturing female Pacific lamprey migrating into the Klamath (Parker et al. 2019), Willamette (Hess et al. 2020), and Columbia (Clemens et al. 2026) Rivers. Through parentage analysis of Snake River Basin larval lamprey, Hess et al. (2022) has also demonstrated that there is variation in maturation timing of Pacific lamprey migrating into the interior Columbia River, with some lamprey delaying spawning for at least a year.

The Clemens et al. (2026) study may unify the two major concepts, providing a possible genetic predisposition for ocean- and stream-maturing life histories that also includes the delayed spawning exhibited by Snake River Pacific lamprey. The data presented suggests that the previous genetic basis for the variation in maturation phenotype (i.e., ocean- vs stream-maturing) is likely a predisposition of relative timing of maturation (i.e., early- vs late-maturing) within each spawning locale (i.e., coastal or interior watersheds) of Pacific lamprey. The genetic basis appears to be a moderate predisposition effect and does not explain the entirety of the decision to complete reproductive development. Before the mechanisms mediating this process can be investigated further, a better understanding of the physiological differences between the early- and late-maturing forms of the interior spawning segment of Pacific lamprey is necessary.

This study aimed to characterize physiological and morphological changes exhibited by the two reproductive life histories (i.e., early- and late-maturing) of Pacific lamprey by monitoring a cohort entering the interior Columbia River (i.e., migrating 234 river kilometers upstream to the first mainstem dam, Bonneville Dam). Within the single migrating cohort of Pacific Lamprey, two divergent life histories were represented: 1) early-maturing fish in which final stages of gonadal maturation complete ~ 10 months after entering freshwater, and 2) late-maturing fish which require an additional year before maturation is complete (i.e., completed maturation ~ 22 months after entering freshwater). To define these two life histories on a physiological level, the current study monitored 99 Pacific lamprey from the 2022 migration year for almost two years, sampling fish monthly, to collect information on their plasma E2 and androstenedione levels, timing of gonad maturation, and morphology of length, weight, and interdorsal fin gap.

## Methods and materials

### Fish and husbandry conditions

Migrating Pacific lamprey were collected via tube trap and collection box at Bonneville Dam on the Columbia River, WA by the Columbia River Inter-Tribal Fish Commission. One third of the lamprey were collected in each of the months of June, July, and August 2022 for a cumulative total of 99 fish. Fish were transported via fish truck (approximately 1325 L of oxygenated river water, with water temperatures maintained between 10 and 20 °C) to the Nez Perce Tribal Hatchery complex on the Clearwater River (Hubbard Gulch, ID; total river kilometer 784). At the first sampling in September 2022, each fish was implanted with a unique 12 mm APT passive inducible transponder (PIT) tag (Biomark; Boise, ID USA). Lamprey were held in covered, rounded square fiberglass tanks (2 m across with a water depth of 0.6 m; approximately 2400 L of water, 24.2 L of water per lamprey) supplied with flowing, seasonally ambient Clearwater River water (high of 16 °C in summer and low of 5 °C in the winter). Tanks were housed in a building with no lighting (i.e., a 0:24 light to dark photoperiod). Lamprey were held at the Nez Perce Tribal Hatchery from their month of collection in 2022 until June 2023. Lamprey characterized as early-maturing were released into the Clearwater River in June 2023.

In June 2023, water availability constraints necessitated moving the 78 lamprey from the group that did not mature (i.e., late-maturing) to the Moscow, ID campus of the University of Idaho. All lamprey were held in a single circular tank (1.5 m in diameter with a water depth of 0.45 m; approximately 795 L of water, 10.2 L of water per lamprey) supplied with aerated, chilled, and dechlorinated City of Moscow water at the College of Natural Resources wet lab. The water supplied to the tank was chilled to temperatures that approximated seasonal ambient conditions (high of 16 °C in summer and low of 8 °C in the winter). Natural light spectrum fixtures were installed within the tank and a simulated natural photoperiod (2500 lx, as measured at the water’s surface) was applied using a timer programed for the latitude (46.732) and longitude (− 117.000) of Moscow, ID. Lamprey were held at the University of Idaho from June 2023 until March 2024. Lamprey characterized as late-maturing (see categorization methods below) were released into the Clearwater River in March 2024.

### Morphology assessments

Once every month all lamprey were anesthetized with sodium bicarbonate-buffered MS-222 (both at 70 mg × L^−1^ water), measured for length (mm), weight (g), and interdorsal fin gap (IDG; mm), To avoid causing undue stress and prevent sampling-associated mortality due to warm water temperatures, the monthly sampling was not conducted during late spring–summer (June – August 2023).

To determine sex and the reproductive development of the gonads (i.e., maturity status), several external cues (i.e., firmness of the abdomen, presence or absence of a post-anal finfold, presence or absence of a urogenital papilla) were assessed at each sampling event (Moser et al. 2019). If lamprey died during the study, a visual assessment of the gonads to establish sex and maturation status was made via necropsy. Beginning in February 2023, a Samsung HM70 EVO ultrasound machine (Samsung; Seoul, South Korea) was added to the monthly sampling workflow to collect ultrasound images of the internal abdominal anatomy, including the gonads, from each fish. Ultrasonography was used to preliminarily determine sex (Kujawa et al. 2019) prior to fish entering the final stages of maturation. Maturation status was categorized based on the expression of external secondary sexual characteristics (see above) and/or gametes in the spring of 2023. Lamprey with soft abdomens, the presence of a post-anal finfold (in the case of females) or a urogenital papilla (in the case of males), and/or expressed gametes at the May 2023 sampling were classified as early-maturing. All other fish were considered late-maturing.

### Endocrinology assessments

At each monthly sampling event, each fish had a 0.9 mL blood sample drawn from the caudal vasculature using a 1 mL heparinized syringe affixed with a 23G × 1″ needle. Blood plasma was immediately separated by centrifugation, snap frozen on dry ice, and stored at − 80 °C until processing. Blood plasma samples were analyzed for E2 and androstenedione. Steroids were extracted from the plasma (50 µL plasma with 150 µL deionized water) twice with diethyl ether, the extracted steroids dried under nitrogen and stored at − 20 °C until being assayed the following week. The lamprey plasma double diethyl ether extraction procedure has been previously validated for E2 (Sower et al. 1983) and androstenedione (Katz et al. 1982). Prior to being assayed, samples were reconstituted in 200 µL deionized water (resulting in a fourfold dilution). Estradiol-17β was measured using a commercial E2 ELISA kit (Cayman Chemical Company; Ann Arbor, MI) following the instructions provided. The assay range for the E2 ELISA is 0.61–10,000 pg/mL, with the lower limit of detection reported as 6 pg/mL. Androstenedione was measured using a commercial androstenedione ELISA kit (IBL International GMBH; Hamburg, Germany) following the instructions provided. The assay range for the androstenedione ELISA is 0.1–10 ng/mL with a lower limit of detection reported as 0.04 ng/mL. Both the E2 and androstenedione ELISA kits were validated for use with extracted Pacific lamprey plasma samples by demonstrating that serially diluted plasma samples from both males and females were parallel to the standard curve of each kit (Online Resource 1). All samples used in the analyses fell within the 20–80% binding range of the standard curves. For a small number of samples collected at the sampling events in May of 2023 and March of 2024, the high plasma E2 levels necessitated that the samples be re-assayed at a higher, eightfold dilution.

### Statistical analysis

As this study was designed to evaluate and establish differences between the sexes (male or female) and maturation life histories (early- or late-maturing), fish were categorized into one of four categories based on a combination of the expression of gametes, the suite of secondary sexual characteristics (described above) present in the last few months prior to spawning, and the ultrasound imagery. These four categories were: early-maturing females (females that were identified as maturing in spring of 2023), early-maturing males (males that were identified as maturing in spring of 2023), late-maturing females (females that did not spawn in spring 2023), and late-maturing males (males that did not spawn in spring 2023). Analyses were conducted to assess differences between these four maturation groups at each sampling event.

Condition factor (CF) was calculated using the following equation, which has been used for both Pacific and sea lamprey (Potter et al. 1978; Whitesel et al. 2020; Youson et al. 1993):

$$CF= \frac{W}{{L}^{3}}\times {10}^{6}$$

where W is weight in grams, L is length in millimeters, and 10^6^ is the constant used for scaling purposes to produce a value close to a single digit. All CF values were then rounded to one decimal.

Prior to performing any analyses, all data at each sampling event were evaluated for normality using both a normal and lognormal distribution. The most prevalent distribution (normal or lognormal) for each sampling event was used in subsequent analyses. When data to be compared were normally distributed, an ANOVA was performed if there were more than two groups to compare (September 2022 through May 2023), followed by Tukey’s multiple comparison post-hoc tests to evaluate differences between groups. When the data was parametric and there were only two groups to compare (September 2023 through March 2024), a T-test was performed. When data were not normally distributed, a Kruskal–Wallis ANOVA was performed if there were more than two groups to compare (September 2022 through May 2023), followed by Dunn’s multiple comparison post-hoc tests to evaluate differences between groups. For non-parametric data with only two groups to compare (September 2023 through March 2024), a Mann–Whitney test to compare ranks was used. Concentrations of both steroid hormones (plasma E2 and androstenedione) were log_10_-transformed prior to any analyses. All analyses were performed using Prism 10.6.0 for macOS (GraphPad Software, LLC). The level of significance was set at *p* < 0.05.

## Results

### Life history definitions

From the initial group of 99 lamprey in September 2022, 21 were early-maturing (10 females, 11 males; 21% of the total). By June 2023 maturation could be determined based on the external secondary sexual characteristics of firmness of the abdomen, presence or absence of a post-anal finfold (in females), presence or absence of a urogenital papilla (in males), and change in IDG over time. For some of these fish eggs or sperm could be manually expressed, confirming their sex and maturity. There was no obvious difference in the pattern of maturity, amongst females or males, relative to the three collection periods of June, July or August at Bonneville Dam. By March 21, 2024, 77 of the remaining 78 late-maturing fish (39 females, 38 males; 78% of the total) exhibited the signs described above indicating that they would reach sexually maturity in the summer of 2024.

### Morphology

**Fig. 1:**
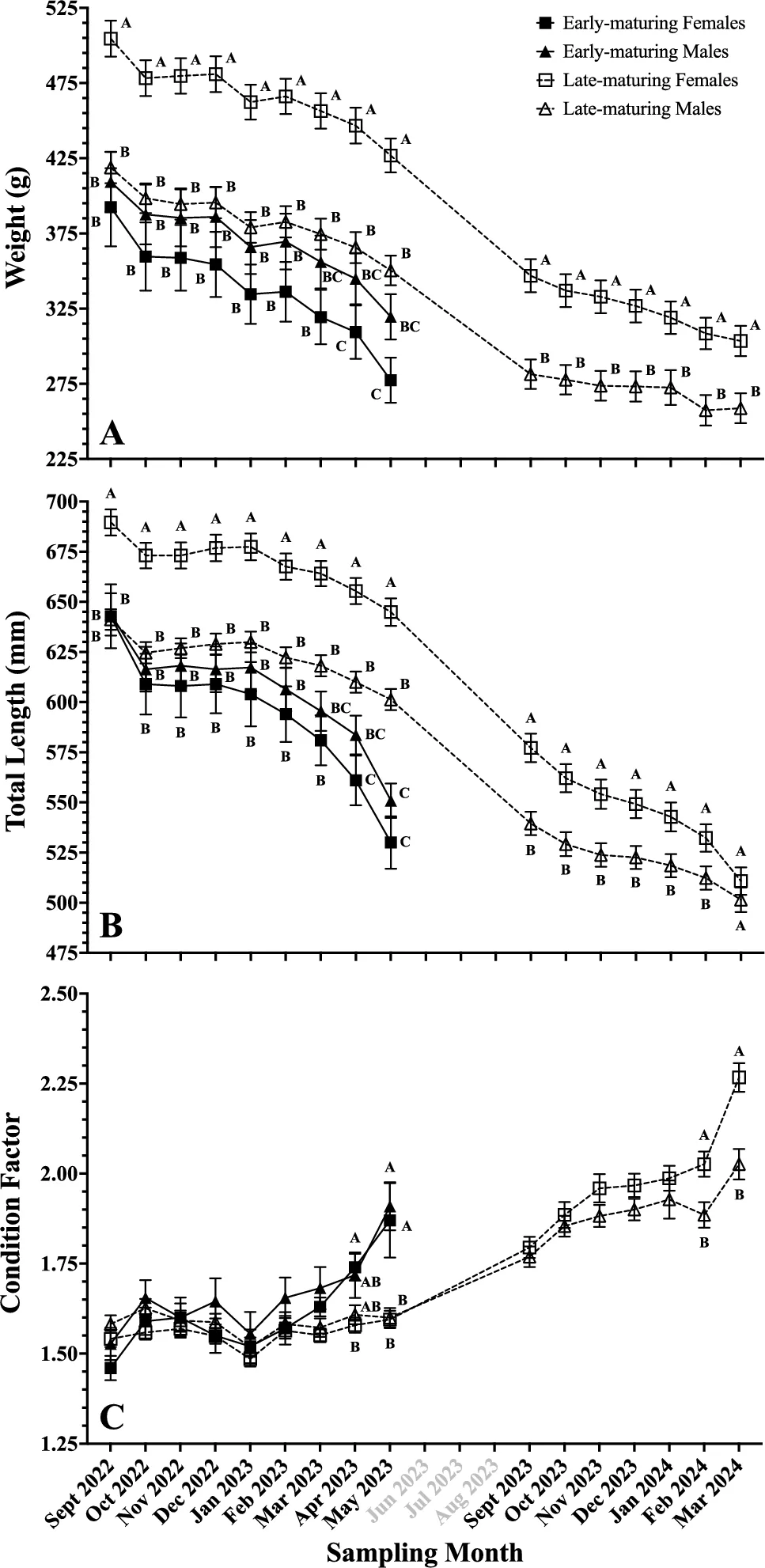
Average weight (panel A), total length (panel B), and condition factor (panel C) ± SEM of Pacific lamprey over the course of the study separated into groups based on their maturation status and sex. Different letters indicate significant differences between groups for respective sampling months (*p* < 0.05 for all comparisons), with letters only being present for sampling months with significant differences between groups. Sample sizes for each group are as follows: late-maturing females = 39, late-maturing males = 39, early-maturing females = 10, early-maturing males, N = 11

The mean weight for each maturation category decreased gradually over the duration of the study (Fig. 1A). At each sampling, the mean weight of the early-maturing females was the lowest and the late-maturing females the highest. From September 2022 through May 2023, the late-maturing female group was always significantly heavier than late-maturing males (*p* < 0.0001), as well as both the early-maturing female and male groups (*p* < 0.001 at all time points). The previous pattern of the late-maturing females being heavier than their male counterparts continued from September 2023 until March 2024 (*p* < 0.01 at all time points).

Like weight, the mean length of all lamprey decreased gradually over time (Fig. 1B). Other than the March 2024 sampling, the mean length of the late-maturing females was always significantly greater than late-maturing males for the duration of the study (*p* < 0.05 at all time points), as well as both early-maturing groups (from September 2022 – May 2023; *p* < 0.01 at all time points). Beginning in March 2023 late-maturing males were significantly longer than early-maturing females (*p* < 0.05 at all time points) and, for May 2023, late-maturing males were also longer than early-maturing males (*p* < 0.001).

Condition factor (Fig. 1C) showed minor change until just prior to spawning in both years. In April 2023, the early-maturing females showed a significant increase compared to the late-maturing females (*p* < 0.05) and in May 2023 the early-maturing males and females were both significantly higher than the late-maturing males and females (*p* < 0.05 for all comparisons). Condition factor increased over time in both sexes in the late-maturation category, with the late-maturing females having a significantly higher CF in February and March of 2024 than the late-maturing males (*p* < 0.01 and 0.0001, respectively).

**Fig. 2:**
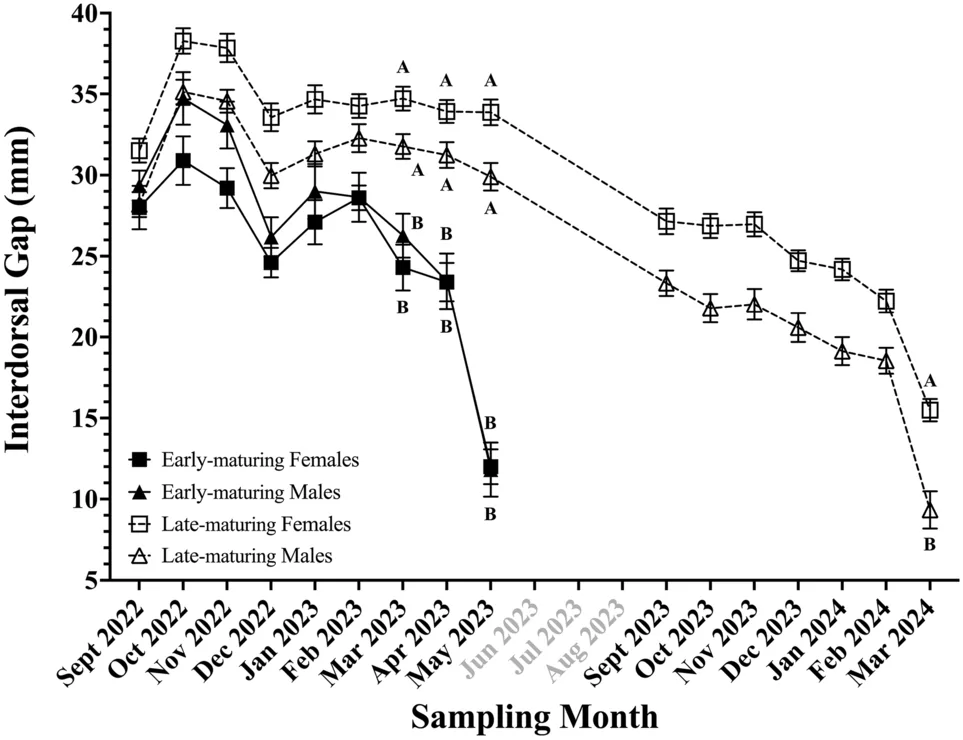
Average interdorsal fin gap (IDG) ± SEM of Pacific lamprey over the course of the study separated into groups based on their maturation status and sex. In March, April, and May of 2023, the late-maturing (unfilled symbols; late-maturing females N = 39, late-maturing males N = 39) and early-maturing (filled symbols; early-maturing females N = 10, early-maturing males N = 11) groups were significantly different from each other (different letters indicate significant differences between groups for respective months; *p* < 0.05 for all comparisons). In March of 2024, the IDG of the late-maturing males and females was significantly different (*p* < 0.01) from the previous month. Letters indicating a significant difference are only present for sampling months where there was a significant difference between groups

The mean IDG showed no consistently significant differences between early-maturing and late-maturing groups until March 2023 (Fig. 2). At the March 2023 sampling timepoint, the mean IDG for both early-maturing females and males became significantly shorter than that for late-maturing females and males (*p* < 0.05 for all comparisons). The decrease in the mean IDG of early-maturing females and males became more apparent in April 2023 (*p* < 0.01 for all comparisons), with the greatest differential between early-maturing and late-maturing lamprey in May 2023 (*p* < 0.001 for all comparisons). The IDG decreased gradually in late-maturing females and males from September 2023 until the lowest level in March 2024, though the sexes were significantly different from each other from September 2023 through the end of the study in March 2024 (*p* < 0.01 at any timepoint).

### Endocrinology

**Fig. 3:**
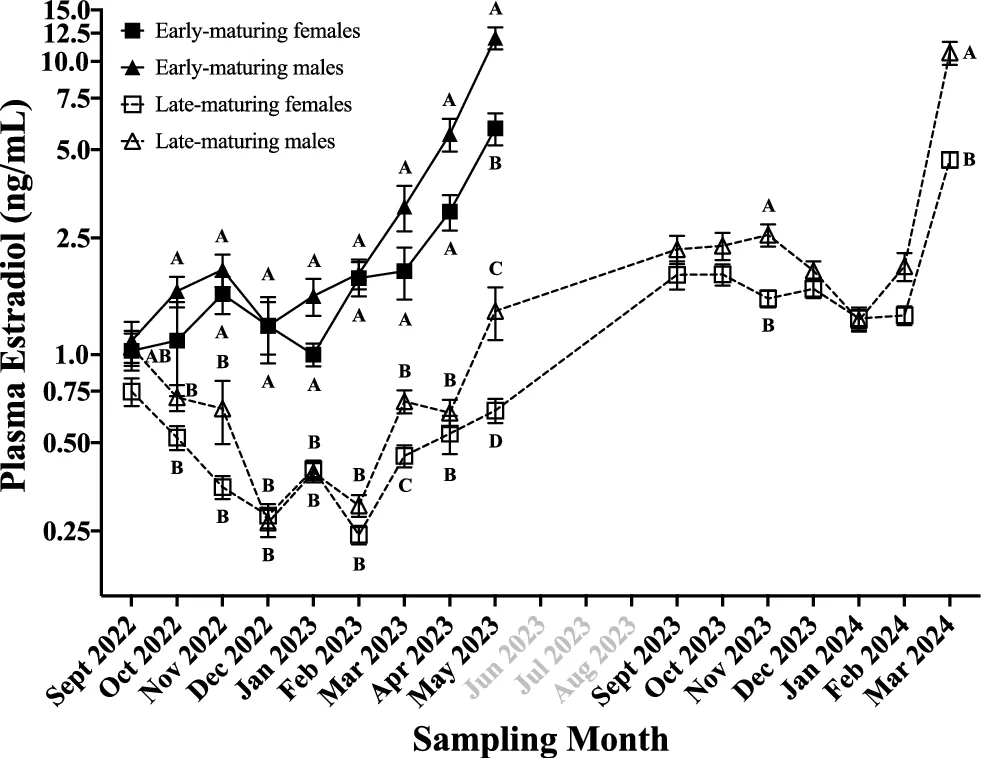
Mean of plasma concentrations of estradiol (E2) ± SEM levels (plotted on a log_10_ scale y-axis) from Pacific lamprey over the course of the study separated into groups based on their maturation status and sex. The sampling events that showed significant differences (*p* < 0.05 for all comparisons) between the sexes and/or maturation groups are indicated by different letters, with letters only being present for sampling months with significant differences between groups. Sample sizes for each group are as follows: late-maturing females = 39, late-maturing males = 39, early-maturing females = 10, early-maturing males, N = 11

Mean plasma E2 (Fig. 3) was higher in males than females for both early-maturing and late-maturing groups throughout the study period (September 2022–March 2024). In the early-maturing male group, mean plasma E2 increased from September 2022 until November 2022, decreased in December 2022, and then increased gradually to the highest level in May 2023. The plasma E2 concentration in early-maturing females initially decreased from September 2022 to October 2022, increased in November 2022, and then decreased slightly in December 2022 and January 2023. Starting at the November 2022 sampling and through the May 2023 sampling, the mean plasma E2 levels in early-maturing males and females was significantly higher than either of the late-maturing groups (*p* < 0.001). After January 2023, mean plasma E2 in early-maturing males and females gradually increased, with the highest levels for these groups measured in May 2023. At the May 2023 sampling point, the mean plasma E2 concentration of early-maturing males was significantly higher than that of early-maturing females (P = 0.0015). Although mean plasma E2 levels in both sexes of late-maturing lamprey were similar to those observed in early-maturing lamprey at the September 2022 timepoint, they decreased to their respective lowest levels measured during the winter period of December 2022 – February 2023. Mean plasma E2 levels of the late-maturing phenotype gradually began to increase in both sexes from March 2023 to May 2023. When sampling resumed in September 2023, mean plasma E2 levels in both sexes of the late-maturing phenotype were slightly elevated compared to the levels measured in the early-maturing groups during the previous fall but remained relatively stable until December 2023. Between February and March of 2024, mean plasma E2 levels in both sexes increased dramatically and by the end of the study (March 2024), E2 levels were the highest levels observed in both sexes of late-maturing lamprey. At the March 2024 sampling point, the mean plasma E2 concentration of late-maturing males was significantly higher than that of late-maturing females *(p* < 0.0001), echoing the sex-based difference seen in the early-maturing lamprey that matured in 2023.

**Fig. 4:**
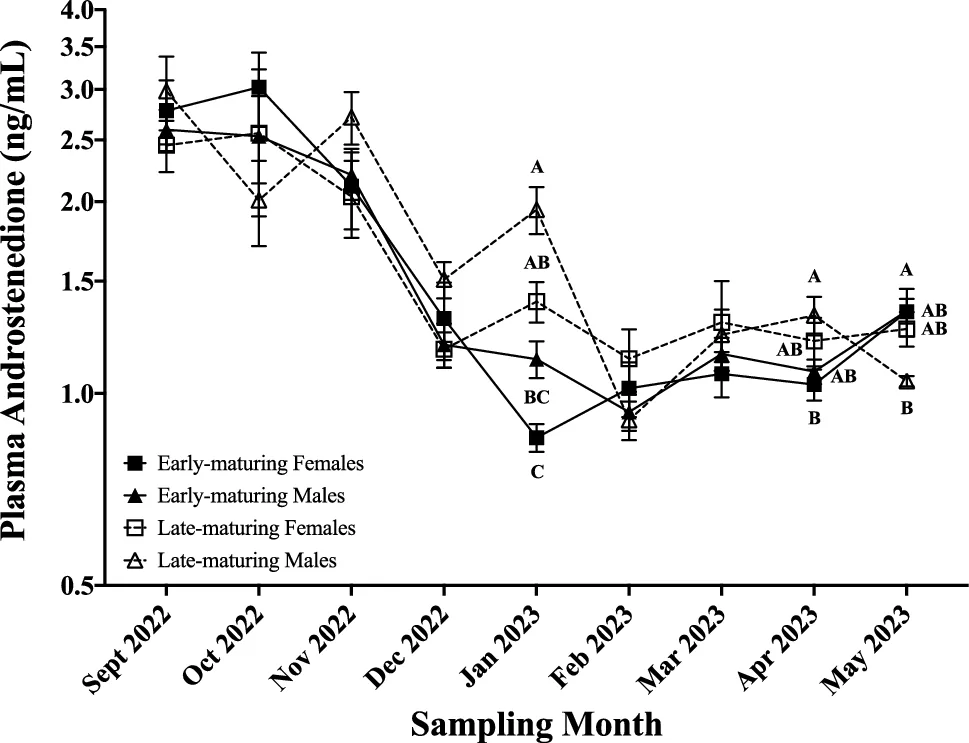
Mean of plasma concentrations of androstenedione ± SEM levels (plotted on a log_10_ scale y-axis) from Pacific lamprey over the course of the study separated into groups based on their maturation status and sex. The sampling events that showed significant differences (*p* < 0.05 for all comparisons) between the sexes and/or maturation groups are indicated by different letters, with letters only being present for sampling months with significant differences between groups. Sample sizes for each group are as follows: late-maturing females = 39, late-maturing males = 39, early-maturing females = 10, early-maturing males = 11

The highest mean plasma androstenedione levels were observed at the first sampling timepoint (September 2022) in both sexes of early-maturing and late-maturing Pacific lamprey (Fig. 4). The plasma concentrations of this steroid generally decreased in all groups from this point until May 2023. In January 2023, the late-maturing males were significantly higher than all other groups *(p* < 0.001) and the early-maturing females were significantly lower than all other groups (*p* < 0.05); however, by the May 2023 sampling, the late-maturing males had significantly lower plasma androstenedione than any other group *(p* = 0.0011) with no other significant contrasts.

## Discussion

Pacific lamprey migrating into rivers along the western coast of North America to spawn are comprised of alternate life histories that diverge based on appearance, migration timing, and the state of gonad development (Beamish 1980; Clemens 2011; Clemens et al. 2016). This study aimed to characterize the morphological and physiological changes associated with alternate life history pathways of Pacific lamprey (early- versus late-maturing) from the interior Columbia River. Reproductive, morphological, and endocrine data, from individually tagged fish serially sampled over many months provide support for two distinct maturation life histories.

The plasma concentrations of E2 significantly increased in both sexes of Pacific lamprey as they approached sexual maturity, like a previous report in this species (Mesa et al. 2010). Similar increases in plasma E2 during sexual maturation and before spawning have been reported in both sexes of sea lamprey (Bolduc and Sower 1992; Linville et al. 1987) and Caspian lamprey (Farrokhnejad et al. 2014). A novel finding in this study was a clear difference in average plasma E2 concentrations between the early- and late-maturing Pacific lamprey groups, months prior to the development of any external morphological differences. Beginning in November 2022, plasma E2 significantly increased in both sexes of early-maturing lamprey as compared to late-maturing lamprey. Plasma E2 levels in early-maturing females and males continued to increase over the winter and spring with the approach of the spawning season in spring 2023, while plasma E2 levels remained low in late-maturing females and males. These results indicate the possibility of using plasma E2 measurement (regardless of sex) as a useful index of reproductive development in Pacific lamprey several months before spawning, a tool that could be useful for artificial propagation efforts (Moser et al. 2019). The overall seasonal pattern and concentration of plasma E2 was comparable between early-maturing and late-maturing lamprey; however, the timing of the E2 increase observed during the last several months varied between the two life histories. While the highest levels of plasma E2 were similar for the two phenotypes, these levels were measured in May in the early-maturing group, whereas the highest levels occurred two months earlier (in March) in the late-maturing group. This could be the result of the life history difference, early- versus late-maturing, or a feature of the different husbandry conditions experienced by the late-maturing group.

As Bryan et al. (2007) identified a specific receptor for androstenedione in the sea lamprey testis, it was theorized that androstenedione might be synthesized by the testis in amounts significant enough to measure in the plasma of male Pacific lamprey – and that these levels would correlate with seasonal reproductive development. Unlike plasma E2 concentrations, there was no increase in plasma androstenedione, in either sex of early-maturing or late-maturing lamprey, that correlated with the progression of reproductive development. These findings, however, do not rule out a paracrine role for androstenedione within the lamprey testis.

It is well known that Pacific lamprey (Beamish 1980), and anadromous lampreys in general (Docker and Potter 2019), do not feed once they leave the ocean and enter freshwater on their spawning migration. They slowly catabolize their body tissues to provide energy (lipid, protein, carbohydrate; Beamish et al. 1979) for migration, avoiding predators, completing gonad development, and spawning (Larsen 1980). A dramatic decrease in body size (weight and length) of both sexes of Pacific lamprey over time was clearly apparent in this study. In contrast, significant changes in CF were not apparent until just prior to spawning in both years. This may be due to the cartilaginous skeleton of lampreys (as opposed to bony fish), which allows for a more uniform reduction of length and mass over time and results in retention of the same general proportions as they get smaller. Several important morphological phenotypic distinctions were observed between fish representing the early-maturing and late-maturing life histories. In general, late-maturing Pacific lamprey of both sexes were larger than either sex of early-maturing lamprey. The significance of the body size difference indicates that late-maturing females and males begin their freshwater spawning migration with more energy than early-maturing lamprey since, once lamprey enter freshwater, the catabolism of their body is the sole source of energy to survive (Whyte et al. 1993). Having more initial energy is potentially an important advantage because late-maturing lamprey spend an additional year in freshwater, not eating, before they spawn.

Another feature of body size was the significant sex difference between late-maturing females and males. Late-maturing females were significantly larger than late-maturing males for most of the study period (September 2022–February 2024). This sex-based, morphological difference supports the idea that the late-maturing females have a higher energetic requirement compared to their male counterparts. Put more specifically, within the late-maturing phenotype, this data implies that the females require more energy than males to support this life history tactic. While most aspects of survival for an extended period in freshwater (approximately 1.5 years) would be similar for female and male lamprey (i.e., evading predators, migration), females require more energy for ovary development than males need to complete formation of mature testes. Previous studies (Porter et al. 2017; Robinson et al. 2009; Whyte et al. 1993) have found the gonadosomatic index (GSI; a ratio of gonad size to body size) of female Pacific lamprey to be more than twice (range = 5–34) that of males (range = 2–7), which (on a mass basis) supports the assertion of a greater energetic need.

Intriguingly, the sex-based difference in body size present in the late-maturing lamprey is not evident for early-maturing female and male Pacific lamprey. In fact, during the period leading up to the completion of reproductive development (September 2022 – May 2023), early-maturing female lamprey were similar (and even slightly smaller) in size compared to maturing male Pacific lamprey. Thus, based on body size, early-maturing females begin their freshwater spawning migration with the least amount of energy compared to early-maturing males and late-maturing females and males. Moreover, the need for larger energy reserves in females (as compared to males), is not apparent in the early-maturing phenotype compared to the late-maturing phenotype. The potential energy limitation that early-maturing female Pacific lamprey experience is likely a strong driver for these females to complete their reproductive cycle at the earliest possible opportunity. Combined with knowledge that late-maturing females from the same migration year are significantly larger and delay maturation longer than early-maturing groups (for at least another year), there would appear to be some size-related component to the migration and maturation processes. While associations of genetic markers (Hess et al. 2014, 2020) with morphological and behavioral trait data (Beamish 1980; Clemens 2011) implies there is a genetic basis underpinning body-size and migration distance, observations of minimal neutral population genetic structure suggests weak philopatry (Hess et al. 2013; Spice et al. 2012). Further study is needed to determine how Pacific lamprey are making the decisions to migrate and complete gonadal maturation, as it seems these are separate decisions which vary depending on the individual.

A decrease in the IDG has been previously reported in sexually maturing Pacific lamprey (Whyte et al. 1993). This study provides further evidence for this secondary sexual character being indicative of maturation, particularly as lamprey get within 2–3 months of full sexual maturation. Both sexes of early-maturing and late-maturing phenotypes exhibited a gradual decrease in IDG in the 6–7 months preceding full gonad development and then a more rapid decrease in the final 1–2 months before spawning. The Pacific lamprey categorized as early-maturing in this study, showed a significant decrease in IDG in March 2023, signaling their rapidly progressing reproductive development. This contrasts with the late-maturing lamprey, which did not exhibit a significant decrease in IDG until the following spring (a year later), signaling their commitment to complete reproductive development. The decrease in the IDG correlates with the gradual reduction in body size that occurs in sexually maturing Pacific lamprey. The significant decrease in length, as these fish catabolize energy reserves in their bodies, results in the shortening of the space between the two dorsal fins. The data in this study confirms the anecdotal evidence that, in the final 1–2 months before potential spawning, the IDG can be combined with other indicators and used as a useful external secondary sexual characteristic that can differentiate early-maturing from late-maturing Pacific lamprey.

In conclusion, the Pacific lamprey in this study clearly demonstrated that the two life histories of early-maturing and late-maturing can be distinguished with morphological and physiological phenotypic data prior to the 10 month mark when the early-maturing fish had fully matured. This was supported by data on timing of reproductive development, changes in morphology of length, weight, and IDG, as well as the profile of plasma concentrations of the sex steroid E2 over time that differed between the two life histories. The plasma E2 results are particularly significant because they signal maturation in Pacific lamprey months before any external morphological indicators. Additionally, while candidate genetic markers for maturation ecotype (i.e., ocean- vs. stream-maturing) show moderate association with gonad-size in female adults from Willamette River, they have not been tested for association with maturation schedule or any proxies of maturation in Pacific Lamprey from the interior Columbia River (Hess et al. 2020). Thus, plasma E2 holds high potential as a physiological indicator to differentiate between these two maturation life histories within the same migration cohort for restoration activities. For translocation, early-and late-maturing fish could be identified and the latter held in captivity until the following year when they will spawn. Plasma E2 concentrations could be used to identify early-maturing fish, suitable for artificial propagation scheduled for the current year, and separate them from late-maturing fish. Evidence of two life histories of Pacific lamprey that migrate into fresh water at the same time but vary the timing of when they spawn (at least a year apart) and complete their lifecycle is intriguing because it is uncommon amongst semelparous anadromous fishes. All semelparous anadromous fishes, studied to date (e.g., salmon), spawn within the year they migrate into fresh water. In addition, as late-maturing lamprey reside in fresh water for almost 2 years without eating, it raises new questions about the indicators driving the decisions to initiate a spawning migration versus completing reproductive development.

## Supplementary Information

Below is the link to the electronic supplementary material.

Supplementary file1 (PDF 192 KB)
