# Supplementary material for: Reproductive and morphological traits distinguish early- and late-maturing Pacific lamprey (Entosphenus tridentatus)
Source: J Comp Physiol B. 2026 Jul 20;196(4):477–86. doi: 10.1007/s00360-026-01692-x (PMC13506653; doi:10.1007/s00360-026-01692-x)
Supplement: Supplementary file 1 [file 360_2026_1692_MOESM1_ESM.pdf]

Supplementary information for the article titled:

Reproductive and morphological traits distinguish early- and late-maturing Pacific lamprey (*Entosphenus tridentatus*)

James J. Nagler*, Lea R. Mederios, Jon E. Hess, Tod Sween, Laurie L. Porter

Journal of Comparative Physiology B

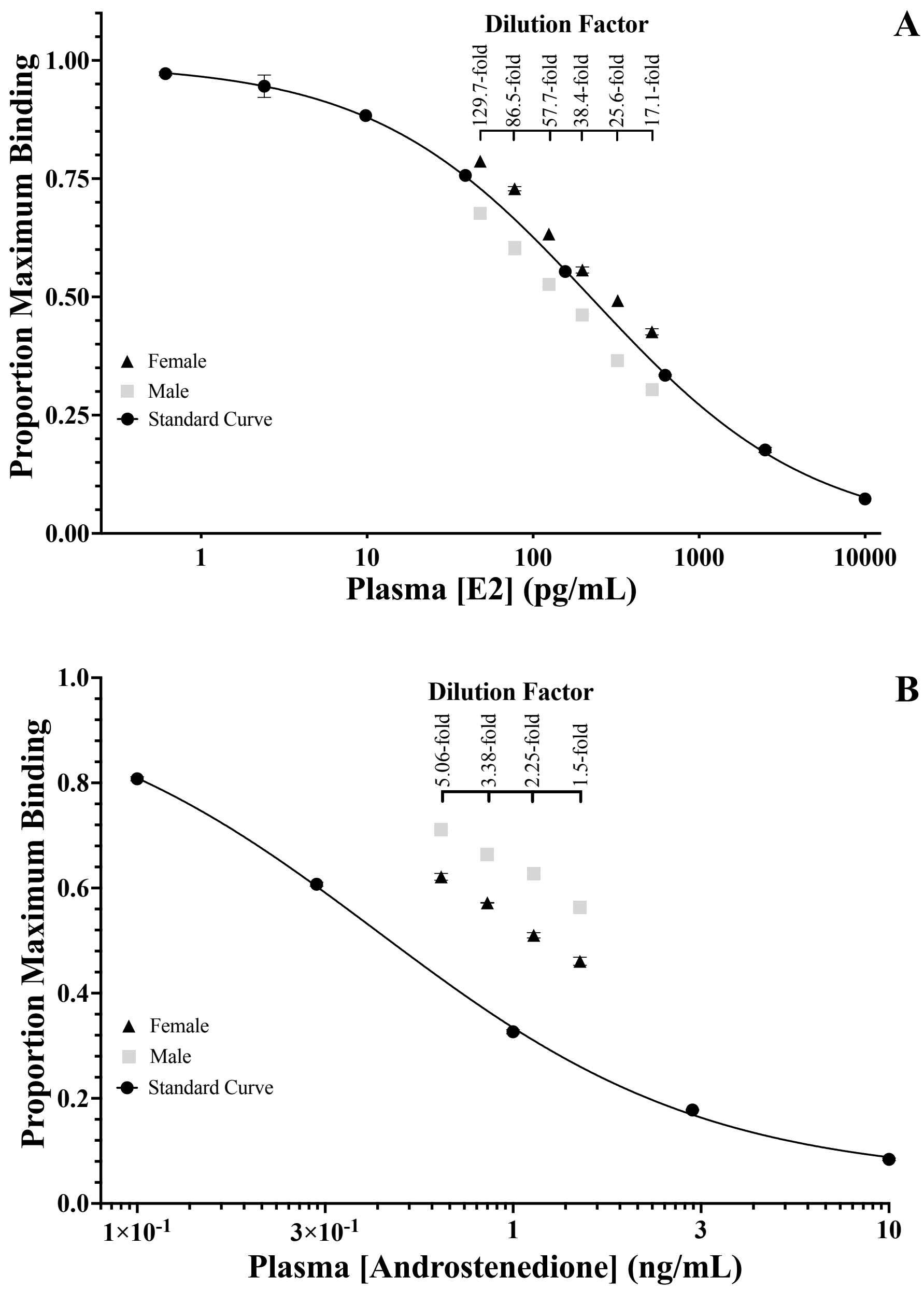

Online Resource 1 Displacement curves demonstrating plasma parallelism between the respective estradiol (A) and androstenedione (B) assay standards and serially diluted female (triangles) and male (squares) Pacific lamprey plasma. Serial dilutions of plasma from male and female Pacific lamprey were parallel to the standard curves for the estradiol ($P = 0.902$ and 0.955, respectively) and androstenedione ($P = 0.941$ and 0.945, respectively) assays.
